# Effects on the estimated cause-specific mortality fraction of providing physician reviewers with different formats of verbal autopsy data

**DOI:** 10.1186/1478-7954-9-33

**Published:** 2011-08-04

**Authors:** Rohina Joshi, Devarsetty Praveen, Clara Chow, Bruce Neal

**Affiliations:** 1 The George Institute for Global Health Australia, Sydney, Australia; 2 The George Institute for Global Health India, Hyderabad, India

**Keywords:** verbal autopsy, questionnaire format, physician reviewer, mortality

## Abstract

**Background:**

The process of data collection and the methods used to assign the cause of death vary significantly among different verbal autopsy protocols, but there are few data to describe the consequences of the choices made. The aim of this study was to objectively define the impact of the format of data presented to physician reviewers on the cause-specific mortality fractions defined by a verbal autopsy-based mortality-surveillance system.

**Methods:**

Verbal autopsies were done by primary health care workers for all deaths between October 2006 and September 2007 in a community in rural Andhra Pradesh, India (total population about 180,162). Each questionnaire had a structured section, composed of a series of check boxes, and a free-text section, in which a narrative description of the events leading to death was recorded. For each death, a physician coder was presented first with one section and then the other in random order with a 20- to 40-day interval between. A cause of death was recorded for each data format at the level of ICD 10 chapter headings or else the death was documented as unclassified. After another 20- to 40-day interval, both the structured and free-text sections of the questionnaire were presented together and an index cause of death was assigned.

**Results:**

In all, 1,407 verbal autopsies were available for analysis, representing 94% of all deaths recorded in the population that year. An index cause of death was assigned using the combined data for 1,190 with the other 217 remaining unclassified. The observed cause-specific mortality fractions were the same regardless of whether the structured, free-text or combined data sources were used. At the individual level, the assignments made using the structured format matched the index in 1,012 (72%) of cases with a kappa statistic of 0.66. For the free-text format, the corresponding figures were 989 (70%) and 0.64.

**Conclusions:**

The format of the verbal autopsy data used to assign a cause of death did not substantively influence the pattern of mortality estimated. Substantially abbreviated and simplified verbal autopsy questionnaires might provide robust information about high-level mortality patterns.

## Introduction

Verbal autopsy methods have their origins in the 17^th ^century lay-reporting systems developed for monitoring epidemics [1]. Those early "death searches" centered upon an interview of the family of the deceased person with the goal of establishing whether the cause of death was attributable to the disease under investigation. During the last 60 years, verbal autopsy methods have evolved in developing countries to allow the broader evaluation of population mortality as well as the study of specific conditions [2]. There are now more than 35 population laboratories in developing-country settings using verbal autopsy methods to track mortality patterns on an ongoing basis [3-5].

While the underlying principles behind the verbal autopsy methodologies used at these sites is the same, the process of data collection and the methods used to assign the cause of death vary significantly [6,7]. It would be anticipated that both these aspects of the verbal autopsy process play an important role in the validity of the causes of death assigned, but robust quantitative evaluations of the impact of using different methods are few [8]. In regard to data collection, a typical verbal autopsy questionnaire collects information about a broad range of characteristics of the deceased including age, sex, education, and occupation, as well as information directly related to the death such as disease risk factors, signs and symptoms of illness, and health-service utilization. Questionnaire format varies markedly with data variously collected in a structured format, free-text format, or combined format. The structured format comprises a list of check boxes and directed responses and the free-text format a narrative description of the illness that led to the death. Assignment of cause of death is usually done by physician review of these data, although expert algorithms and data-driven algorithms are increasingly widely used [6]. Once again, a broad range of different cause of death assignment processes are in use [9,10].

In this study, we sought to determine the impact of the format of the verbal autopsy data on the cause-specific mortality fractions reported for a rural community in Andhra Pradesh, India, using a verbal autopsy process based upon single-physician review.

## Methods

This study was conducted by a research collaboration (the Andhra Pradesh Rural Health Initiative) [11] involving five Indian and Australian institutions. The data used for this analysis were collected between Oct. 1, 2006 and Sept. 30, 2007. Ethics approval for the project was received from the Ethics Committees of the CARE Foundation, Hyderabad, India; the Indian Council of Medical Research, New Delhi, India and the University of Sydney, Australia. Informed consent was obtained from each respondent prior to the collection of any data, and we sought to design and conduct the project in line with the Declaration of Helsinki and its subsequent amendments. For participants who could not read or write, the participant information sheet and consent form were explained by the Multipurpose Primary Healthcare Worker (MPHW) and a thumbprint was recorded.

### Population studied

This project was conducted in 45 villages in the East and West Godavari districts in Andhra Pradesh, India. The population (n = 180,162) age and sex structure was defined by a population census conducted in 2002-2003 [12]. The age distribution of the population in the villages was characteristic of populations in which fertility has decreased recently, with relatively low proportions of the population in the very young and very old age groups. A quarter of the population was below 15 years of age and a tenth was above 60 years of age. The majority of the adult population were engaged in work related to agriculture or aquaculture and the average household income was 2,000 Indian rupees (US$50) a month. The literacy level of the population was 54% [11].

### Identification of deaths

The primary responsibility for identifying all deaths in the village lay with the MPHW. Identification of deaths by the MPHW was facilitated by her daily contact with the villagers and a network of key informants including the village headman, the "Panchayat" (village governing body responsible for registration of deaths), priests and cremation staff, and other community leaders.

### Data collection

For each death recorded, the MPHW attempted to visit the deceased's household within a month of the date of death. The family member or other caregiver best able to report on the events preceding the death was identified, consent was obtained, and a systematic inquiry into the events leading up to the death was made using a verbal autopsy tool. The verbal autopsy tool used in this project had two sections. The first section was composed of a series of structured questions beginning with a filter question for each symptom group to allow skipping past more detailed questions that were not likely to be relevant to the death. The second free-text section recorded an open-ended narrative documenting the history of the illness leading to death as described by the family member. This free-text section was completed with the aid of a defined symptom list with specific inquiry about treatments, medical procedures, and associated documentation. Different questionnaires were used for deaths in each of three age groups (0 to 28 days, 29 days to less than five years, and five years onward). The questionnaires were based on validated verbal autopsy tools used by studies in China [13] and Tanzania [14] and the Registrar General of India's Sample Registration System [15] with minor modifications to terminology made to suit local circumstances. The MPHWs were trained in data collection prior to commencement of the study and were provided with a manual of operations developed specifically for the administration of the questionnaire. Refresher training was provided every six months.

### Cause of death assignment

Cause of death assignment was done by single-physician review using validated materials and processes developed for the Registrar General of India's Sample Registration System [4,15]. This included providing the physician coders, who had received specific training in verbal autopsies, a series of algorithms to facilitate the cause of death assignment process [16]. In this study, the process was modified such that the information obtained for each death was presented to the same physician coder three times, each time in a different format, with a cause of death assigned independently on each occasion. In brief, for each recorded death, the physician coder was, at random, presented first with either the structured data alone or the free-text data alone and asked to assign a cause of death. After an interval of 20 to 40 days, the data were presented again in the alternate format and a cause of death was assigned a second time. Finally, after another 20 to 40 days, the full data for the death (composed of the structured and free-text sections together) was presented and an index cause of death was assigned. On each occasion, a single underlying cause of death was selected for each individual from a restricted list of causes drawn from the 10^th ^version of the International Classification of Disease (ICD-10).

### Outcomes

The main outcome for this study was the proportion of deaths that were attributed to each of 15 main causes (defined at the chapter heading level in the ICD-10) or else remained unclassified.

### Analysis

The primary analysis was a comparison of the cause-specific mortality fractions and rank order of the leading causes of death ascribed to the population using cause of death assignment based on the structured data alone, the free-text data alone, and the combined data (with the index cause based on using the two formats of data together). The proportions of deaths assigned to each main cause (or left unclassified) were presented graphically side by side in a column chart to enable a direct visual comparison of the cause-specific mortality fractions described by the structured, free-text, and combined methods.

At the level of the individual case, kappa statistics and their confidence intervals were calculated to quantify the consistency of reporting of cause of death between each of the three pairs of methods. This was done in two ways - first, using the full 16 possible assignments to obtain an overall estimate of the correlation of diagnoses and, second, for each individual cause compared to all others to identify whether it was possible to conclude whether particular causes were more or less likely to have consistent diagnoses made. For the second set of analyses, an indicator variable was created in each dataset and set as 1 if the diagnosis was the cause of interest and 0 if the diagnosis was any other cause or unclassified. Analyses were done using SPSS version 16 [17].

## Results

During the 12 months of the study, a total of 1,497 deaths were recorded, of which 1,407 (94%) had a verbal autopsy completed. There were structured and free-text data available for all 1,407 cases for which a verbal autopsy was done. There was a slightly greater proportion of unclassified deaths (ICD chapter R00-R99) when assignment was based on the free-text data format alone (21.6%) or structured data format alone (19.3%) compared to when the two sets of data were used together (15.4%).

### Overall pattern of mortality

**Figure 1:**
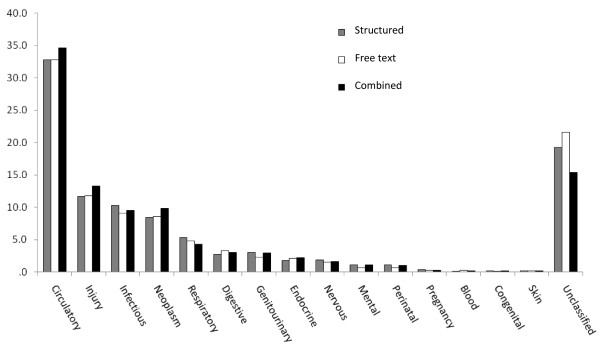
**The proportion of deaths assigned to each main underlying cause for each of the different data formats presented to the physician coders**.

The cause-specific mortality fractions described for this population varied little with the format of the data presented to the physician coders (Figure 1). The rank order of the leading causes of death was almost identical for cause of death assignment based on the structured, free-text, and combined data formats with only small differences in the estimated cause-specific mortality fractions. While neoplasms ranked fourth in the structured and free-text format but third in the combined format, the differences in the proportions of deaths assigned to these main causes among data formats was small and may simply reflect the play of chance.

While the cause-specific mortality fractions varied little with the format of the data used by the physician for cause of death assignment, there was moderate variation in the causes of death assigned to individuals after each presentation of the data (Table 1). The causes of death assigned using the structured format matched the index in 1,012/1,407 (72%) of cases with a kappa statistic of 0.66 (95% confidence interval [CI]: 0.63, 0.68). For the free-text format, the corresponding figures were 989/1,407 (70%) and 0.64 (95% CI: 0.61, 0.67).

**Table 1:** Kappa statistics describing constancy of individual cause of death assignments between different data formats presented to the physician coders

Cause of death (number of deaths)	Kappa statistics (95% confidence interval)		
	
	Structured vs. combined format	Unstructured vs. combined format	Structured vs. unstructured
Circulatory (487)	0.71 (0.67 - 0.75)	0.70 (0.66 - 0.74)	0.57 (0.52 - 0.61)
Injury (187)	0.81 (0.76 - 0.86)	0.77 (0.72 - 0.82)	0.74 (0.68 - 0.79)
Infectious (134)	0.75 (0.69 - 0.80)	0.70 (0.63 - 0.76)	0.65 (0.58 - 0.72)
Neoplasm (139)	0.83 (0.78 - 0.88)	0.79 (0.73 - 0.84)	0.76 (0.70 - 0.82)
Respiratory (61)	0.57 (0.47 - 0.67)	0.57 (0.47 - 0.68)	0.48 (0.38 - 0.58)
Digestive (43)	0.38 (0.24 - 0.51)	0.52 (0.40 - 0.65)	0.36 (0.23 - 0.50)
Genitourinary (42)	0.53 (0.40 - 0.66)	0.61 (0.48 - 0.74)	0.36 (0.22 - 0.50)
Endocrine (31)	0.53 (0.37 - 0.69)	0.55 (0.40 - 0.70)	0.31 (0.15 - 0.48)
Nervous (23)	0.61 (0.44 - 0.77)	0.63 (0.46 - 0.80)	0.37 (0.19 - 0.55)
Mental (16)	0.62 (0.42 - 0.82)	0.46 (0.22 - 0.70)	0.53 (0.30 - 0.77)
Perinatal (14)	0.83 (0.68 - 0.98)	0.66 (0.44 - 0.88)	0.48 (0.23 - 0.72)
Pregnancy^# ^(4)			
Blood^# ^(3)			
Congenital^# ^(3)			
Skin^# ^(3)			
Unclassified (217)	0.44 (0.38 - 0.50)	0.40 (0.34 - 0.46)	0.31 (0.25 - 0.37)
Overall (1407)	0.66 (0.63 - 0.68)	0.64 (0.61 - 0.67)	0.53 (0.50 - 0.56)

*A kappa statistic of 0.75 or above is generally considered to reflect a high correlation, 0.40 - 0.75 a moderate correlation, and below 0.40 a poor correlation [21]. ^#^No kappa coefficient was calculated because there were too few deaths were recorded to allow informative estimates to be obtained.

Further examination of the main causes of death shows that correlations between individual diagnoses were moderate or high for most causes when comparing either format to the index. Correlations were particularly good for the four leading causes (circulatory, injury, infections, and neoplasms) but appeared consistently lower for the next four leading causes (respiratory, digestive, genitourinary, and endocrine) although confidence intervals were not tight. For the remaining seven causes, there were too few cases assigned to each to enable reliable estimates of kappa coefficients. The overall correlation (0.53, 95% CI: 0.50, 0.56) was lower for the comparison of structured versus free text as were the great majority of the cause-specific estimates made for this comparison. This is unsurprising because there was no overlap of data when assigning the causes of death for this comparison, whereas for each of the comparisons against the index cause the comparator data format was also used to help allocate the index cause of death.

## Discussion

The two key findings from this project are that neither the format of the data presented to the physician reviewer, nor the joint provision of data in two different formats versus a single format alone had any substantive impact on the cause-specific mortality fractions estimated for the population. The cause-specific mortality fractions obtained from the provision of each data format, or the two combined, were highly comparable in every case with no substantive differences detected for any major cause. These findings are somewhat in conflict with usual practice, with the majority of verbal autopsy programs collecting both structured and free-text data in the belief that the combined data will provide physician reviewers with important additional insight into the likely cause of death [7,8], perhaps because the two different formats of questioning are complementary to one other [10]. On the basis of the results reported here, it seems possible that simplified approaches could provide much the same information. It may be that if the cause of death cannot be assigned on the basis of one format of data alone, then the cause is so unclear that the addition of further information will assist in making a diagnosis in too few cases for it to be important. Indirectly, the results also suggest that if such profoundly different questionnaire formats can give such similar results there is probably rather little to be gained from making minor adjustments to existing questionnaires and that the real advances in verbal autopsy methodology are to be made elsewhere.

The findings are important because the format of the questionnaire has a significant influence on many aspects of the verbal autopsy process. Most importantly of all, if the questionnaire need only collect data in one format, it could be substantially reduced in length, avoiding the duplication of data collection consequent upon having a structured and free-text-narrative component to an interview [18]. This would make the verbal autopsy interview both more acceptable to the respondents and more feasible for those collecting the data. The restriction of data collection to only structured questions would produce even greater dividends because complexity would be reduced. Interviewers collecting free-text-narrative data require much more extensive training and supervision and must have at least a basic understanding of disease processes and the pattern in which symptoms develop [6]. For a completely structured questionnaire, no such understanding is required and this would expand the base from which interviewers could be recruited and reduce the average cost of an interview. Structured questions also make it much easier to collect standardized data across a range of interviewers without specialization [10] and reduce the likelihood that important aspects of the events leading to death will be missed.

There are also several possible disadvantages to a switch to a fully structured questionnaire. First, the questionnaire could miss important information if the scope of the questions is inadequate, and unless very long it will always miss unusual signs or symptoms associated with uncommon causes of death. This will not be an issue for the evaluation of broad mortality patterns in populations, but if verbal autopsy is required to identify particular, less-common conditions then additional specific questions might need to be added. The list of structured questions in the verbal autopsy tool used for this study was fairly exhaustive and included a number of questions about health care utilization and investigation results that are not always covered by the structured sections of verbal autopsy questionnaires. This is likely a key reason for the success with which causes of death were assigned using the structured data alone in the current study. Another potential challenge with fully structured questionnaires is that the structured format can impede the ability of the interviewer to develop a rapport with the respondent and that the structured format is at odds with the way that medical education teaches practitioners to collect and review clinical information [19]. Careful design of a structured questionnaire and the interview flow, or a switch to automated probabilistic methods of cause of death assignment would, however, address these issues.

While the findings of this research are in contrast to usual practice in the conduct of verbal autopsy projects, the results are not totally unexpected, since no prior attempt has been made to address this issue in a robust quantitative design. Prior work done as part of the Andhra Pradesh Rural Health Initiative has shown that another well-established practice in verbal autopsy, the duplicate coding of cases by physicians, is no more effective than single coding [20]. These two pieces of work serve to highlight the importance of robust quantitative evaluation of all aspects of verbal autopsy design to ensure that the most effective and efficient systems are in place.

The analyses done for this study were based on a fairly short cause list (chapter headings of ICD10) and this will have decreased the potential for misclassification. While the list of causes used here would be sufficient for many high-level health-planning or program-monitoring functions, a longer cause list may have increased the variability of the cause-specific mortality fractions estimated with each data format and likely would have decreased the strengths of the correlations obtained at the individual level. It is likely that our use of the same physician to make all three cause of death assignments has overestimated the correlation among the causes assigned using the different data formats because some cases may have been remembered from one cause of death assignment to the next. The alternate strategy of using different physicians to assign each cause would almost certainly have done the opposite because it would have introduced between-physician variability. We undertook the study in this way in an effort to specifically test the impact of the data format while holding all other factors constant. Nonetheless it has to be recognized that the current results are likely to be biased toward, rather than away from, congruence in the findings for the two data formats. Repeating the study using different physicians would nicely answer this question. It is also possible that the conclusions drawn here may not be fully generalizable to all other settings in which verbal autopsy is done. In part at least, the findings are likely to be dependent upon both the specifics of the methods used in this study and the true cause-specific mortality fractions in the population.

## Conclusions

The results of this study suggest that the format of the verbal autopsy data used to assign a cause of death does not substantively influence the pattern of mortality estimated, and that the collection of data in both structured and free-text formats is probably unnecessary. This conclusion is supported by other work showing that the inclusion of free-text data in probabilistic cause of death assignment models had no appreciable effect. This also provides support for the notion that automated cause of death assignment processes might have considerable potential for the reliable allocation of cause of death - computer-based, machine-learning techniques more easily utilize data in structured formats and the research presented here suggests that rather little information would be lost by forgoing the free-text component of data collection. These findings have substantial implications for the design and implementation of future verbal autopsy studies that seek to describe the mortality pattern in a community. The data suggest that abbreviated and simplified verbal autopsy questionnaires could provide robust information for functions such as health-service planning, program evaluation and the long-term tracking of cause-specific mortality fractions.

## Competing interests

The authors declare that they have no competing interests.

## Authors' contributions

RJ, CC, and BN participated in the conception, design, and coordination of the study. RJ and DP performed the statistical analysis and drafted the paper. All authors read, contributed to, and approved the final manuscript.
